# Tofacitinib in Steroid-Refractory Acute Severe Ulcerative Colitis: A Retrospective Analysis

**DOI:** 10.7759/cureus.45416

**Published:** 2023-09-17

**Authors:** Sayan Malakar, Srikanth Kothalkar, Umair Shamsul Hoda, Uday C Ghoshal

**Affiliations:** 1 Gastroenterology, Sanjay Gandhi Postgraduate Institute of Medical Sciences, Lucknow, IND

**Keywords:** herpes zoster virus, cytomegalovirus, steroid refractory ulcerative colitis, salvage therapy, tofacitinib, steroid-refractory ulcerative colitis, inflammatory bowel disease, acute severe ulcerative colitis

## Abstract

Introduction: Steroid-refractory acute severe ulcerative colitis (ASUC) patients are at the highest risk of colectomy. Among the available options, cyclosporine and infliximab have similar efficacy but infliximab is a costly drug and cyclosporine has multiple side effects like kidney injury, neurotoxicity, and dyselectrolytemia. Surgical management is often associated with higher morbidity. Newer oral small molecules like Janus kinase inhibitors are the ideal molecules to bridge the gap. Tofacitinib has already been extensively evaluated in patients with moderate to severe UC; however, data on ASUC treated by tofacitinib are limited.

Methods: We retrospectively analyzed the data of patients with ASUC who were admitted to our hospital’s luminal gastroenterology unit between January 2021 and July 2023. Patients with ASUC who were managed with tofacitinib were included in the study.

Results: Eight patients with ASUC were identified who did not respond to intravenous hydrocortisone and were treated with tofacitinib. The mean age was 39 ± 15 years and 87.5% were female. The median duration of illness was 24 months (interquartile range (IQR): 12-120 months). Seven of eight patients (87.5%) responded to oral tofacitinib 10 mg twice a day by the fifth day of treatment. The median follow-up period was six months (IQR: 1-12 months). One patient required colectomy and one patient had varicella zoster reactivation requiring treatment discontinuation.

Conclusion: Tofacitinib is an attractive alternative to the currently available salvage therapy for steroid-refractory ASUC; however, long-term efficacy and risk remain to be explored.

## Introduction

Acute severe ulcerative colitis (ASUC) is a medical emergency that requires hospitalization and steroid therapy [1]. Of patients with ulcerative colitis (UC), 15-25% present with ASUC. Patients who fail to respond to steroids in seven days have a one-year colectomy rate of 54% [2]. The options for salvage therapy in steroid-refractory ASUC include cyclosporine, infliximab, and surgery [3]. However, in resource-constraint settings, biological therapy is not always feasible, and hypoalbuminemia is strongly associated with poor response to infliximab [4]. Surgery in this setting is also not devoid of complications and morbidities, so oral small molecules like tofacitinib are helpful in bridging the gap [5]. Emerging evidence suggests that tofacitinib can be used in patients with ASUC [6]. Here, we present our experience with tofacitinib in patients with ASUC.

## Materials and methods

We have retrospectively analyzed the data of all ASUC patients who were admitted to our luminal gastroenterology unit between January 2021 and July 2023. All patients were diagnosed with ASUC based on Truelove and Witt’s criteria [1]. ASUC is diagnosed when a patient has a bloody stool frequency of ≥ six along with any of these systemic features such as pulse rate > 90 per minute, hemoglobin < 10.5 g/dL, C-reactive protein (CRP) > 1 mg/dL, erythrocyte sedimentation rate > 30 mm per hour, or a body temperature of >37.8°C. Patients underwent complete baseline investigations, electrolytes including potassium, magnesium, erect X-ray abdomen, CRP, fecal calprotectin (FCP), proctosigmoidoscopy with biopsy for histopathological examination (HPE), and cytomegalovirus (CMV) infection and toxin assays for *Clostridium difficile* infection (CDI) [2]. Endoscopic disease activity was assessed using the Mayo and Ulcerative Colitis Endoscopic Index of Severity (UCEIS) scores [7]. Patients were started on intravenous (IV) hydrocortisone 100 mg IV every six hours and low molecular weight heparin for thromboprophylaxis. Patients with higher CMV burden in the colon were treated with IV ganciclovir for five days, followed by oral valganciclovir 900 mg twice a day for the next two to three weeks [8]. In addition to that, the workup for biologicals and small molecules was also started with hepatitis B surface antigen (HBsAg), anti-hepatitis C virus (HCV) antibody, a total core antibody against hepatitis B, contrast-enhanced computed tomography (CECT) of the chest, interferon-gamma release assay (IGRA), and lipid profile [9]. Clinical disease activity was assessed on day three of IV steroids using the Oxford criteria [10]. According to these criteria, after three days of IV steroids, patients who had persistent bloody stool frequency of more than eight or stool frequency of three to eight along with CRP > 45 mg/dL were considered for salvage therapy. Patients who did not respond to three to five days of steroid therapy were started on infliximab (IFX), cyclosporine, or oral tofacitinib (10 mg twice a day) after ruling out any contraindications [11]. Patients were regularly monitored for cytopenia, reactivation of latent infections, liver injury, and hyperlipidemia [9]. The response was defined as a reduction of the Mayo score [12]. Clinical response was defined as the reduction of baseline Mayo score by ≥3 points and a decline of 30% from the baseline score with a decrease of at least one point in rectal bleeding score subscore or an absolute rectal bleeding subscore of 0-1. Clinical remission was defined by a Mayo score of ≥2 with no individual score of >1. In long-term follow-up, endoscopic healing was considered if the mucosal subscore was 0-1 [12]. Follow-up FCP, CRP, and colonoscopy were performed after 12 weeks of therapy.

## Results

From our luminal gastroenterology unit’s database, eight patients of steroid-refractory ASUC were identified who were treated with tofacitinib. The mean age of presentation was 39 ± 15 years and most of them were female (87.5%). The median duration of illness was 24 months (interquartile range: 12-120 months). Their baseline features of severity are described in Table 1.

**Table 1:** Patients' demographic, baseline severity parameters, response to the treatment, side effects profile, and outcome Table showing patients' baseline demographic, clinical, biochemical, and endoscopic features. All patients were started on tablet tofacitinib 10 milligrams twice a day. Treatment-related outcomes and adverse events are also reported. Seven of eight patients responded and one patient required a colectomy. The patient died after colectomy. Another patient developed bacterial pneumonia and died after one month. Herpes zoster infection was seen in one patient requiring discontinuation of the therapy. 5-ASA: 5-amino salicylate; CMV: cytomegalovirus.

Features	Patient 1	Patient 2	Patient 3	Patient 4	Patient 5	Patient 6	Patient 7	Patient 8
Age	27	23	30	55	34	54	34	55
Sex	Female	Female	Male	Female	Female	Female	Female	Female
Extent (Paris)	E3	E2	E3	E3	E3	E3	E3	E3
Duration (years)	4	2	1	10	12	2	1	20
Treatment experience	5-ASA experienced, azathioprine experienced, steroid-refractory	Steroid refractory, infliximab + vedolizumab experienced, tacrolimus intolerant, azathioprine experienced	5-ASA experienced, azathioprine naïve, biological naive	Steroid refractory, 5-ASA experienced, azathioprine naive	Steroid-refractory, azathioprine defaulter, no finances	Steroid-dependent, azathioprine-induced cytopenia	Steroid nonresponsive	Steroid responsive but intolerant, azathioprine-induced cytopenia, defaulter
Bloody stool frequency/day	12	6	6	8	8	7	10	7
C-reactive protein (<1 g/dL)	52	2.1	85	78	30	9	41	18
Albumin (3.5-5.5 g/dL)	2.8	4.2	1.9	2.2	2.1	3.2	1.7	3.7
Hemoglobin (13-16 g/dL)	9.2	11.7	7.9	10.4	9.8	11.9	7.1	9.1
Fecal calprotectin (<50 mcg/g stool)	1282	736	2531	1562	1536	899	2916	1298
Mayo endoscopic score	2	3	3	2	2	3	3	3
Ulcerative Colitis Endoscopic Index of Severity (UCEIS)	4	6	6	4	4	6	5	6
CMV copies/25 mg colonic tissue	11000	3275	3640	24800	4 x 10^9^	Negative	Negative	Negative
Clostridium difficile toxin	Culture and toxin negative	Culture and toxin negative	Culture and toxin negative	Culture and toxin negative	Culture and toxin negative	Toxin positive, culture negative	Culture and toxin negative	Not available
Tofacitinib dose	10 mg twice a day	10 mg twice a day	10 mg twice a day	10 mg twice a day	10 mg twice a day	10 mg twice a day	10 mg twice a day	10 mg twice a day
Response days to respond	Responded 5 days	Responded 4 days	Responded 3 days	No response	Responded 5 days	Responded 3 days	Responded 5 days	Responded 4 days
Follow-up period	16 months	12 months	1 month	10 days	4 months	12 months	1 month	9 months
Side effects	Nil	Hyperlipidemia	Nil	Nil	Nil	Herpes labialis (after four days of therapy), dose reduced	Bacterial pneumonia, died after one month	Developed herpes zoster after five days of therapy, discontinued
Colectomy/death	No/No	No/No	No/No	A colectomy was done. The patient developed sepsis after the surgery and died	No/No	No/No	No/died of bacterial pneumonia	No/No

**Figure 1:**
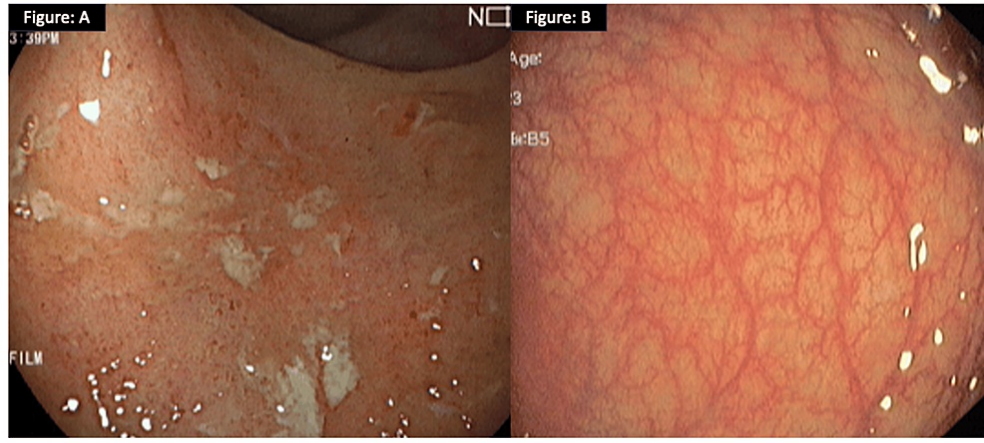
Proctosigmoidoscopy findings of the patient with acute severe ulcerative colitis On presentation with acute severe ulcerative colitis, proctosigmoidoscopy of patient 1 (A) showed multiple ulcerations with loss of vascular pattern (partial Mayo score: 2) in the rectum. After three months of therapy with tofacitinib, the patient's symptoms resolved. Repeat proctosigmoidoscopy revealed a completely healed mucosal pattern in the rectum suggestive of a partial Mayo score of 0 (B).

All of them had severe disease (Mayo endoscopic score: 3, UCEIS: 5-6, hypoalbuminemia, and anemia). None of them had toxic megacolon. In our series, all patients were 5-amino salicylate (ASA) experienced, four (50%) patients were azathioprine experienced, two discontinued azathioprine because of cytopenia, one was azathioprine defaulter, and another was azathioprine naive. Only one patient was infliximab and vedolizumab experienced. They received IV hydrocortisone for five to seven days before adding tofacitinib. The median follow-up period was six months (IQR: 1-12 months). Following tofacitinib, seven out of eight patients (87.5%) responded to the therapy and all of them responded within five days of initiation of the therapy (Figure 1).

One patient did not respond after seven days of tofacitinib therapy. In view of worsening bloody diarrhea, tofacitinib was stopped and she required subtotal colectomy. On postoperative day seven, the patient died of peritonitis and sepsis.

Side effects were noted in four patients. All of them were on 10 mg twice-a-day therapy. Varicella zoster virus (VZV) reactivation was seen in one patient after five days and herpes labialis in one patient after four days of therapy leading to discontinuation of tofacitinib. One patient succumbed to bacterial pneumonia after a month of tofacitinib therapy. The dose was reduced to 5 mg twice daily in a patient who developed dyslipidemia after three months of therapy.

In our series, two patients (25%) were started on tofacitinib instead of IFX because they could not afford biologicals. Both responded to tofacitinib.

## Discussion

Steroid-refractory ASUC patients are at higher risk of colectomy and tofacitinib is one of the promising options. Biological therapy is costly and surgical management is associated with significant morbidity. In our study, 87.5% of patients responded to tofacitinib treatment, and one patient died following colectomy. Similar reports from India have also shown that tofacitinib can be used as salvage therapy in ASUC though robust data are lacking. There are a few case series and a systematic review showing the pooled efficacy of tofacitinib is 75% as the first-line therapy [13]. In most series, patients were steroid and biological experienced. As the second and third line of therapy, its efficacy ranges from 67% to 85% [6,14,15]. The efficacy of tofacitinib in anti-tissue necrosis factor (TNF)-experienced UC is also promising, with colectomy-free survival approaching 80% [16]. One of our patients was tacrolimus intolerant and infliximab, adalimumab, and vedolizumab experienced; she also responded to tofacitinib. The role of tofacitinib in vedolizumab-experienced UC is still unclear and requires more data. In a study by Berinstein et al. [17], the remission rate was even higher with tofacitinib compared to vedolizumab in patients with anti-TNF experienced UC. However, the study did not include patients with ASUC.

The incidence of VZV reactivation ranges from 4.1% to 10% depending on the dose and the duration of the therapy whereas dyslipidemia occurs in 10% of patients [11,18]. A serious bacterial infection was seen in a patient leading to death. Cases of *Pneumocystis jirovecii*-related pneumonia have been reported in patients with rheumatoid arthritis on tofacitinib [19]; however, bacterial infection can also complicate the course of the disease because of immune paralysis. In our series, none had venous thromboembolism or other complications. There is also a theoretical risk of CMV reactivation in patients with Janus kinase 2 inhibitors [20]. Four of eight patients had baseline higher CMV copies in rectal tissue; however, none of them had relapsed following tofacitinib therapy.

Also, tofacitinib is a cheaper option for UC patients compared to biologicals. A previous study from India has shown a higher financial burden among patients with active inflammatory bowel disease compared to patients who are in remission [21]. The median annual cost for the treatment was ₹75,146 (49,447-92,212) and ₹52,436 (49,229-67,567) for patients who had relapsed in Crohn’s disease and UC, respectively. In that study, the median salaries of their earning family members were ₹ 10,000-14,500 per month. The annual cost of IFX treatment is around ₹300,000 in India, which is beyond the affordability range for most of the middle to lower-income group patients. For them, tofacitinib is an alternative cost-effective option. Similar findings were also shown in a recent study from Japan [22]. For moderate to severe UC, tofacitinib is a cost-effective option compared to the biologicals.

This is one of the largest series of ASUC patients managed with tofacitinib. Limitations of the study include the absence of long-term follow-up and its retrospective model. Vaccination for VZV was not done, as the new recombinant vaccine was not available in India and live ones are contraindicated. Colonic CMV load was not assessed following therapy as they were in remission, so the effect of tofacitinib on the colonic CMV load could not be assessed. Despite its limitations, our study has shown that tofacitinib can be an alternative to IFX in steroid-refractory ASUC. Larger data with long follow-ups are required to investigate steroid-free remission and colectomy-free survival.

## Conclusions

In conclusion, tofacitinib can be an excellent choice for steroid-refractory patients with ASUC. It is an oral small molecule and does not require therapeutic drug monitoring. The growing level of evidence suggests hypoalbuminemia is an independent predictor of non-responsiveness to infliximab in UC. Tofacitinib is an attractive cost-effective alternative. It has a fast onset of action as all of the patients responded by the fifth day of treatment in our series. The only drawback with tofacitinib treatment is adverse drug reactions, which include hyperlipidemia and VZV reactivation. Careful selection of patients and routine follow-up is warranted. A multicenter randomized controlled trial is necessary to address its long-term safety and efficacy.
